# Activation of NLRP3 by uropathogenic *Escherichia coli* is associated with IL-1β release and regulation of antimicrobial properties in human neutrophils

**DOI:** 10.1038/s41598-020-78651-1

**Published:** 2020-12-14

**Authors:** Isak Demirel, Alexander Persson, Annelie Brauner, Eva Särndahl, Robert Kruse, Katarina Persson

**Affiliations:** 1 iRiSC - Inflammatory Response and Infection Susceptibility Centre, Faculty of Medicine and Health, Örebro University, Örebro, Sweden; 2 School of Medical Sciences, Örebro University, Campus USÖ, 701 82 Örebro, Sweden; 3 Division of Clinical Microbiology, Department of Microbiology, Tumor and Cell Biology, Karolinska Institutet and Karolinska University Hospital, Stockholm, Sweden; 4 Department of Clinical Research Laboratory, Faculty of Medicine and Health, Örebro University, Örebro, Sweden

**Keywords:** Cellular microbiology, Antimicrobial responses, Immune evasion, Infection

## Abstract

The NLRP3 inflammasome and IL-1β have recently been linked to the severity of uropathogenic *Escherichia coli* (UPEC)-mediated urinary tract infection (UTI). However, not much is known about the contribution of NLRP3 to the antimicrobial properties of neutrophils and the release of IL-1β during UPEC infection. The purpose of this study was to elucidate the mechanisms behind UPEC-induced IL-1β release from human neutrophils, and to investigate the contribution of the NLRP3 inflammasome in neutrophil-mediated inhibition of UPEC growth. We found that the UPEC strain CFT073 increased the expression of NLRP3 and increased caspase-1 activation and IL-1β release from human neutrophils. The IL-1β release was mediated by the NLRP3 inflammasome and by serine proteases in an NF-κB-and cathepsin B-dependent manner. The UPEC virulence factors α-hemolysin, type-1 fimbriae and p-fimbriae were all shown to contribute to UPEC mediated IL-1β release from neutrophils. Furthermore, inhibition of caspase-1 and NLRP3 activation increased neutrophil ROS-production, phagocytosis and the ability of neutrophils to suppress UPEC growth. In conclusion, this study demonstrates that UPEC can induce NLRP3 and serine protease-dependent release of IL-1β from human neutrophils and that NLRP3 and caspase-1 can regulate the antimicrobial activity of human neutrophils against UPEC.

## Introduction

Urinary tract infection (UTI) is one of the most common infections in humans. It is known that approximately 30% of all women will have at least one episode of UTI by the age of 24, and 60% will have at least one UTI during their lifetime^1^. Uropathogenic *Escherichia coli* (UPEC), expressing an array of virulence factors crucial for host adherence, nutrition acquisition, mobility and modulation of host immune responses^2,3^, is the predominant cause of UTI^4,5^. One fourth of women with UTI will have a recurring infection within six months, which leads to exaggerated consumption of antibiotics. This in combination with the increased number of multidrug-resistant UPEC isolates have made treatment options limited^6^. Understanding the host–pathogen interaction and the mechanisms behind a successful colonization of the urinary tract by UPEC, could provide valuable clues in the search for alternative treatment strategies.

During UTI, release of chemokines, e.g. IL-8, CXCL1 and CXCL2, cause recruitment of neutrophils to the superficial epithelium and the bladder lumen to target the infection^7,8^. Within two hours after infection, the first neutrophils are found in the urinary tract of mice, and the influx of neutrophils has been found to be proportional to the bacterial load^9^. Neutrophils are the main effector cells of the innate immune response during UTI, and they are critical for UPEC clearance^8^. Activated neutrophils can kill UPEC through reactive oxygen species (ROS), antibacterial granular agents, phagocytosis and neutrophil extracellular traps (NETs)^10–13^. Mice with defective neutrophil response have been shown to be more susceptible to UTI, and they have a decreased ability to clear the infection^9^. Thus, neutrophils are critical for the bacterial clearance during UTI, but they are also largely associated with the tissue cytotoxicity observed during the infection. The tissue damage mediated by neutrophils is linked to production of ROS and other cytotoxic products that neutrophils release in the urinary tract^14^.

IL-1β is one of the most potent pro-inflammatory cytokines and it has been linked to dysregulated inflammation and to the severity of the UTI^15–20^. The inflammasomes, which are cytosolic multiprotein complexes, detect danger/damage signals and/or bacterial virulence factors and activate caspase-1, which leads to the maturation and release of IL-1β and pyroptosis (cell death). It has recently been shown that UPEC can activate the nod like receptor pyrin domain containing 3 (NLRP3) inflammasome in bladder epithelial cells by α-hemolysin^15,17^. The activation of this inflammasome usually requires two signals. The initial priming affects NLRP3 and pro-IL-1β at the mRNA transcription level and signal two mediates the assembly of the NLRP3 inflammasome that results in a subsequent caspase-1 activation^21,22^. UPEC has been found to prevent NLRP3 complex formation by the TLR-signaling inhibitory protein (TcpC)^16^. This could give UPEC the ability to regulate the activation of the NLRP3 inflammasome, i.e. as a virulence strategy to subvert the host immune system. It appears that the signaling pathways for maturation of IL-1β in neutrophils differ from pathways activated in e.g. epithelial cells and monocytes/macrophages. In neutrophils, IL-1β release has been shown to involve activation of inflammasomes and of different serine proteases, such as proteinase 3, neutrophil elastase and cathepsin G^23–26^. The role of IL-1β in the progression of UPEC-induced UTI is unclear and the results from the literature are contradictory. While one study has reported that mice lacking IL-1β are protected from UTI^20^, other studies have shown that UPEC-induced IL-1β is part of the innate immune response that limits and controls the colonization of the urinary tract^16,27^.

Although most studies investigating the role of inflammasomes and IL-1β during UTI have been conducted on macrophages and bladder epithelial cells^15,17,28^, several reports suggest that neutrophils also are a major source of IL-1β during infections^23,25,26^. *Staphylococcus aureus *^23,29^, *Streptococcus pneumonia*^30^ and *Salmonella typhimurium*^31^ infections have all been shown to be able to induce inflammasome-mediated caspase-1 activation and subsequent IL-1β maturation in neutrophils. The aim of the present study was to elucidate the mechanism behind UPEC-induced IL-1β release from neutrophils, and to investigate the contribution of the NLRP3 inflammasome to the antimicrobial activities of neutrophils.

## Material and methods

### Neutrophil isolation

Human neutrophils were isolated from healthy blood donors by density gradient centrifugation of Polymorphprep and Lymphoprep reagents (AXIS-SHIELD PoC AS, Oslo, Norway) or with the EasySep Human Neutrophil Enrichment Kit (STEMCELL Technologies, Cambridge, UK) according to the manufacturer’s instructions^32^. The cells were infected in RPMI 1640 (Lonza, Basel, Switzerland) complemented with 10% heat inactivated fetal bovine serum (FBS) during the experiments. An ethical approval has been granted by the regional ethics review board in Uppsala, Sweden (Dnr 2015/437), to isolate blood from healthy individuals after informed consent. Blood from healthy donors were collected according to the ethical guidelines of both the Declaration of Helsinki and the Swedish national board of health and welfare. The viability of the neutrophils was > 90% as determined by the trypan blue exclusion test after isolation.

### Bacterial strain and mutants

The UPEC strain CFT073 was used in the experiments. CFT073 deletion mutants CFT073Δ*hlyA* and CFT073Δ*fimH* were made with the λ red recombinase system^33^ using primer sets *hlyA*_Fwd 5′-AAAAACAAGACAGATTTCAATTTTTCATTAACAGGTTAAGAGATAATTAAGTGTAGGCTGGAGCTGCTTC-′3 and *hlyA*_Rev 5′-AATCTTATGTGGCACAGCCCAGTAAGATTGCTATTATTTAAATTAATAAAATGGGAATTAGCCATGGTCC-′3 and primer sets *fimH*_Fwd 5′- CATTCAGGCAGTGATTAGCATCACCTATACCTACAGCTGAACCCAAAGAGGTGTAGGCTGGAGCTGCTTC -′3 and *fimH*_Rev 5′- TAGCTTCAGGTAATATTGCGTACCTGCATTAGCAATGCCCTGTGATTTCTATGGGAATTAGCCATGGTCC -′3, respectively (Thermo Fisher Scientific, Waltham, MA, USA)^15^. The CFT073Δ*pap* isolate^34^ was a kind gift from Professor Harry Mobley at the University of Michigan. The CFT073Δ*TcpC* isolate was a kind gift from Professor Catharina Svanborg at Lund University. The bacteria were grown in Lysogeny broth (Lennox, Franklin Lakes, NJ, USA) overnight at 37 °C on a shaker in an aerobic milieu.

### Stimulation of neutrophils

Human neutrophils were stimulated with the wild type CFT073 or CFT073 mutant bacteria for 3 or 6 h at a multiplicity of infection (MOI) of 1 or 10 and incubated at 37 °C with 5% CO_2_. Neutrophils were also pre-incubated with caspase-1/4 inhibitor AC-YVAD-CHO (10 µM, Enzo Life Sciences, NY, USA), caspase-3 inhibitor AC-DEVD-CHO (10 µM, Enzo Life Sciences), NLRP3 inhibitor MCC950 (2 µM, Avistron Chemistry Services, Cornwall, UK), JNK inhibitor SP600125 (10 µM, InSolutionT M JNK Inhibitor II, Calbiochem, USA), p38 MAPK inhibitor SB203580 (10 µM, Santa Cruz Biotechnology Inc., Heidelberg, Germany), ERK1/2 inhibitor PD98059 (10 µM, Santa Cruz Biotechnology Inc), NF-κB inhibitor BAY 11–7082 (5 µM, Enzo Life Sciences), serine protease inhibitor 3,4-Dichloroisocoumarin (DCI, 100 µM, Merck Millipore, MA, USA), cathepsin B inhibitor CA074 (100 µM, Apexbio Technology LLC, Houston, USA), actin polymerization inhibitor cytochalasin D (Cyto D, 10 µg/ml, Santa Cruz Biotechnology Inc), receptor-interacting serine/threonine-protein kinase 3 (RIPK3) inhibitor GSK-872 (10 µM, R&D Systems, Minneapolis, USA) or DMSO as vehicle control, for 1 h prior to CFT073 stimulation for 3 or 6 h at MOI 1 or MOI 10^15^. mRNA, proteins and supernatants were collected and kept at − 80 °C until further analysis.

### RNA isolation and real time RT-PCR

Total RNA was isolated from neutrophils using E.Z.N.A. Total RNA Kit I (Omega Bio-tek, GA, USA) according to manufacturer’s instructions. The RNA yield was determined using spectrophotometry (Nano-Drop ND-1000, Wilmington, NC, USA). First strand cDNA synthesis was performed by High capacity cDNA RT kit (Thermo Fisher Scientific). The real time-RT-PCR was performed with Maxima SYBR Green qPCR Master Mix (Thermofisher), 10 ng cDNA and 250 nM of each primer (Supplementary Table S1, designed by Origene (Maryland, USA) and synthesised by Eurofins MWG Synthesis GmbH (Ebersberg, Munich)). The PCR amplification was conducted in a CFX96 Touch Real-Time PCR Detection System (Bio-Rad Laboratories, Hercules, CA, USA) using the following protocol: initial denaturation at 95 °C for 10 min, 40 cycles of denaturation at 95 °C for 15 s followed by annealing/extension at 60 °C for 60 s. The mRNA expression was analyzed by the comparative Ct (ΔΔCt) method and normalized to the endogenous control GAPDH. Fold difference was calculated as 2^−ΔΔCt^^15^.

### Measurement of IL-1β release and cell viability

IL-1β release from neutrophils was analyzed by an enzyme-linked immunosorbent assay (ELISA). IL-1β was measured using the human IL-1β kits (ELISA MAX Deluxe Sets, BioLegend, San Diego, CA, USA) and the optic density (OD) was evaluated with a spectrophotometer (Multiskan Ascent, Thermo Labsystems, Helsingfors, Finland). Cell viability was evaluated by Pierce lactate dehydrogenase (LDH) cytotoxicity assay (Thermo Fisher Scientific) according to the manufacturer’s protocol^15^.

### Western blot analysis

Neutrophils were harvested in RIPA buffer supplemented with Halt protease and phosphatase inhibitor cocktail (Thermo Fisher Scientific). The cells were homogenized using a syringe and needle. The protein concentration of the samples was measured with the DC protein assay (Bio-Rad Laboratories). Equal amounts of sample were mixed with Laemmli buffer and boiled for 5 min in 95 °C. Furthermore, neutrophil supernatants were precipitated with 10% Trichloroacetic acid (TCA, Merck Millipore) for 1 h on ice followed by acetone wash before the pellet was re-suspended in 4 × Laemmli buffer (Sigma-Aldrich, St. Louis, MO, USA) and boiled for 10 min in 95 °C. The samples (10 µg or total supernatant precipitate) were separated by 4–20% SDS-polyacrylamine gel electrophoresis and transferred to a polyvinylidene fluoride (PVDF) membrane (Bio-Rad Laboratories). The PVDF membrane was blocked with 3% BSA for 1 h. Caspase-1 protein was detected using a mouse monoclonal (AdipoGen Life Sciences, Buckingham, UK) against human caspase-1. NLRP3 was detected using a mouse monoclonal (Abnova, Taipei City, Taiwan) against human NLRP3. GAPDH was detected with a rabbit polyclonal antibody (Santa Cruz Biotechnology Inc). All primary antibodies were incubated overnight. As secondary antibodies, goat anti rabbit IgG (horseradish peroxidase, HRP) (Abcam, Cambridge, UK) and goat anti mouse IgG (HRP) (Abcam) were used and incubated for 1 h at room temperature. The blots were developed using Luminata Forte Western HRP Substrate (Merck Millipore)^15^.

### Measurement of total ROS-production

A luminol-horseradish peroxidase (HRP) assay was used to measure total reactive oxygen species (ROS)-production from neutrophils. Neutrophils in RPMI 1640 containing 10% FBS were incubated with luminol (0.1 mg/mL, Sigma Aldrich) and HRP (4 U/mL, Roche, Basel, Switzerland) for 15 min at 5% CO_2_ and 37 °C^35^. Neutrophils were then pre-incubated with DMSO (vehicle), caspase-1/4 inhibitor AC-YVAD-CHO (10 µM) or NLRP3 inhibitor MCC950 (2 µM) for 1 h prior to CFT073 stimulation at MOI 10 and 37 °C. The luminescence was measured in a microplate reader (Fluostar Optima, BMG Labtech, Aylesbury, UK) every third minute during 2 h.

### Measurement of bacterial growth

Neutrophils in RPMI 1640 containing 10% FBS were pre-incubated at 5% CO_2_ and 37 °C with DMSO (vehicle), caspase-1/4 inhibitor AC-YVAD-CHO (10 µM) or NLRP3 inhibitor MCC950 (2 µM) for 1 h prior to CFT073 stimulation at MOI 0.1. The absorbance was measured with the Cytation 3 plate reader (BioTek, Winooski,VT, USA) at 600 nm every 10 min during 8 h. The absorbance of only neutrophils was measured and subtracted from the absorbance of the co-incubated samples (bacteria + neutrophils). The relative growth inhibition (delta OD600) was calculated as absorbance of (bacteria + DMSO) − (absorbance of bacteria + neutrophils)^36^.

### Phagocytosis assay

Phagocytosis was evaluated by pre-incubated neutrophils in RPMI 1640 containing 10% FBS with DMSO (vehicle), caspase-1/4 inhibitor AC-YVAD-CHO (10 µM) or NLRP3 inhibitor MCC950 (2 µM) for 1 h prior to CFT073 (carrying an eGFP-plasmid, enhanced green fluorescence protein, kindly provided by Professor Philip Poole at University of Oxford, UK) stimulation at MOI 10 for 3 h. The neutrophils were then washed twice with PBS to remove non-phagocytosed bacteria. Phagocytosis was quantified by measuring the mean florescence intensity of the phagocytized CFT073 (eGFP) bacteria using the Gallius (Beckman Coulter, Brea, USA) flow cytometer with 488 nm laser and FL1 525/40 nm band-pass filter. The data was analyzed with Kaluza Flow Cytometry Analysis v1.3 (Beckman Coulter)^36^.

### Data analysis

Data are expressed as mean ± SEM. Differences between groups were evaluated by one-way ANOVA followed by Bonferroni multiple testing correction. Differences were considered statistically significant at p < 0.05. n = number of independent biological replicates.

## Results

### Expression of inflammasome-related genes

**Figure 1:**
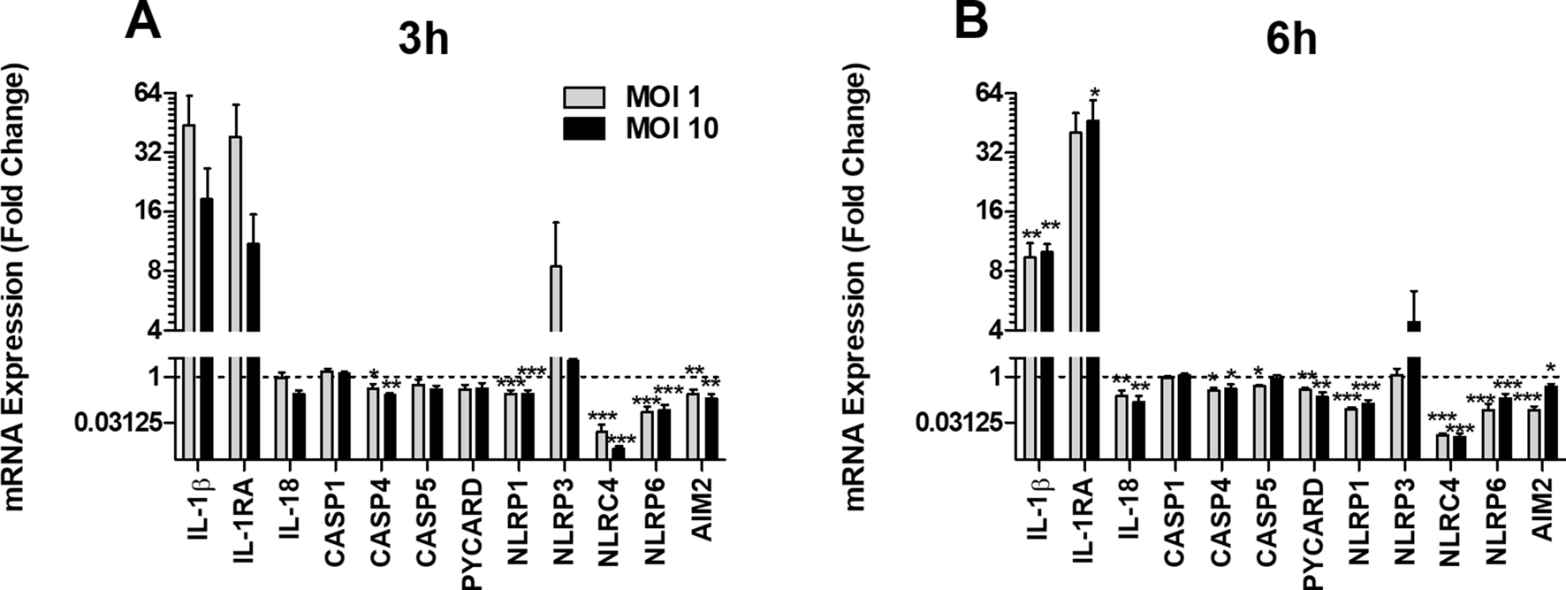
Gene expression of inflammasome-related genes. Human neutrophils were infected with UPEC strain CFT073 at MOI 1 or MOI 10 for 3 h (**A**) and 6 h (**B**) followed by analysis of mRNA expression. The gene expression was normalized to GAPDH and expressed as fold change relative to unstimulated controls. Data are presented as mean ± SEM (n = 3 independent experiments) with the y-axis as Log2. Bars below 1 (the dotted line) indicate decreased gene expression. Asterisks denote statistical significance compared to respective unstimulated control (*p < 0.05, **p < 0.01, ***p < 0.001).

Human neutrophils were infected with UPEC and the gene expression of inflammasome-related proteins was assessed. UPEC significantly decreased the gene expression of *CASP4*, *NLRP1*, *NLRC4*, *NLRP6* and *AIM2* at MOI 1 and MOI 10 after 3 h of infection compared to unstimulated cells (Fig. 1A). After 6 h, the gene expression of *IL-1β* and *IL-1RA* was significantly upregulated by UPEC, while a decreased expression of *IL-18, CASP4*, *CASP5*, *PYCARD*, *NLRP1*, *NLRC4*, *NLRP6* and *AIM2* was observed in stimulated compared to unstimulated cells (Fig. 1B). Notably, the gene expression of *CASP1* or *NLRP3* was not significantly altered by UPEC after 3 or 6 h. Taken together, these findings show that UPEC alters the expression of several inflammasome-related genes in neutrophils.

### Caspase-1 activation and IL-1β release

**Figure 2:**
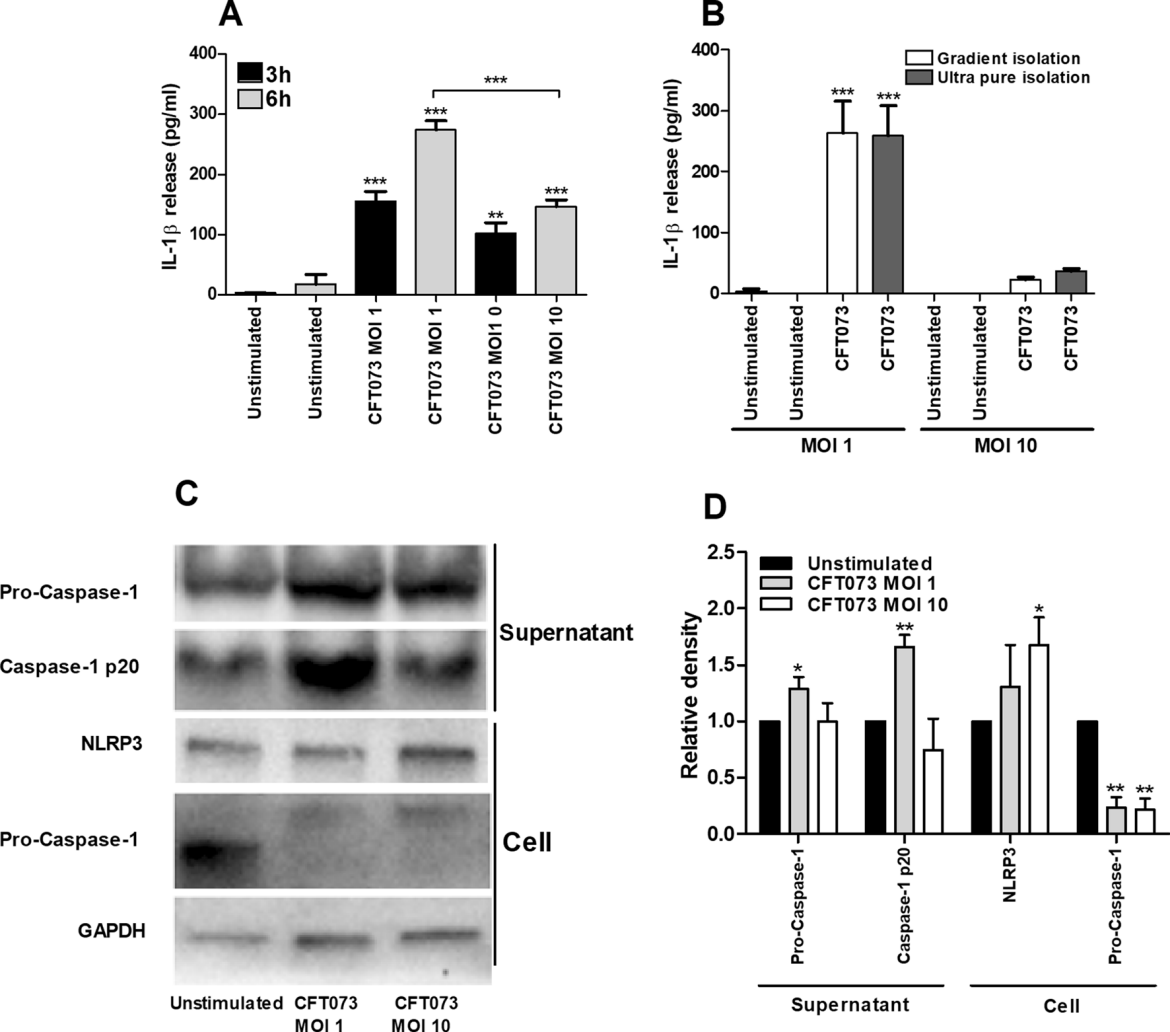
IL-1β release and protein expression of caspase-1 and NLRP3. Neutrophils were infected with UPEC strain CFT073 at MOI 1 or MOI 10 for 3 h (**A**) and 6 h (**A**–**D**) followed by analysis of IL-1β release (**A**,**B**) and caspase-1 and NLRP3 protein expression by Western blot analysis (**C**,**D**). GAPDH was used as a loading control and to normalize the quantification of the Western blots. Original western blot presented is available in Supplemental Fig. S1. Data are presented as mean ± SEM (n = 3 independent experiments). Asterisks over bars denote statistical significance compared to respective unstimulated control (*p < 0.05, **p < 0.01, ***p < 0.001).

We continued with evaluating the ability of UPEC to induce IL-1β release and activation of caspase-1 in human neutrophils. UPEC caused a significant increase in IL-1β release at MOI 1 and MOI 10 after 3 and 6 h compared to unstimulated cells, with a significantly higher release at MOI 1 than at MOI 10 after 6 h (Fig. 2A). In order to rule out the possibility of monocyte contamination as the source of IL-1β in our samples, control experiments were performed comparing gradient-isolated neutrophils compared to beads isolated (ultra-pure) neutrophils. However, there was no difference in IL-1β release from either isolation method upon UPEC infection after 6 h to support such a contamination (Fig. 2B). Western blot analysis demonstrated an increased p20 (active) form of caspase-1 and pro-caspase-1 in the cell supernatant upon UPEC stimulation for 6 h compared to unstimulated cells. Increased protein levels of NLRP3 were found in the cell fraction after UPEC infection compared to unstimulated cells (Fig. 2C,D).

### The role of NLRP3, caspase-1 and serine proteases

**Figure 3:**
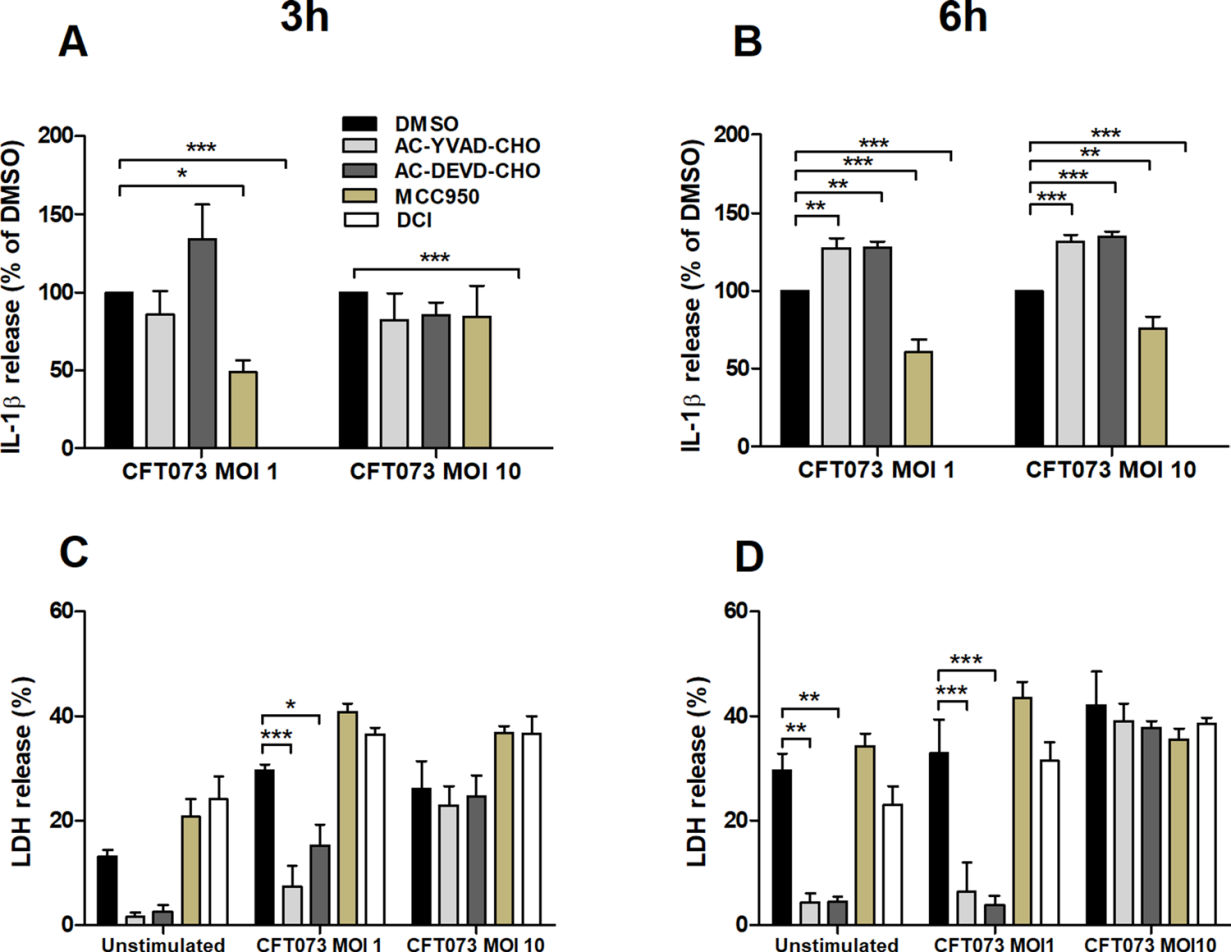
NLRP3 and serine proteases are associated with UPEC-induced IL-1β release. Neutrophils were pre-incubated with DMSO (vehicle), caspase-1/4 inhibitor AC-YVAD-CHO (10 µM), caspase-3 inhibitor AC-DEVD-CHO (10 µM), NLRP3 inhibitor MCC950 (2 µM) or serine protease inhibitor 3,4-Dichloroisocoumarin (DCI, 100 µM) for 1 h prior to UPEC strain CFT073 infection at MOI 1 or MOI 10 for 3 h (**A**,**C**) and 6 h (**B**,**D**) followed by analysis of IL-1β (**A**,**B**) and LDH release (**C**,**D**). LDH release is presented as % of total LDH. Data are presented as mean ± SEM (n = 3 independent experiments). Asterisks denote statistical significance (*p < 0.05, **p < 0.01, ***p < 0.001).

To further evaluate the role of NLRP3, caspase-1 as well as serine proteases in UPEC-induced IL-1β release, pharmacological inhibitors were employed. Treatment of neutrophils with the NLRP3 inhibitor MCC950 or the serine protease inhibitor DCI significantly reduced UPEC-induced IL-1β release compared to cells subjected to the vehicle control DMSO (Fig. 3A,B). In contrast, the caspase-1/4 inhibitor AC-YVAD-CHO and the caspase-3 inhibitor AC-DEVD-CHO significantly increased IL-1β release after 6 h compared to cells subjected to the vehicle control DMSO (Fig. 3B). The classic NLRP3 inflammasome activation pathway leads to both IL-1β release and pyroptosis, and studies on neutrophil cell toxicity (LDH release) were performed to investigate a possible association between IL-1β release and pyroptosis in neutrophils. However, the NLRP3 inhibitor or serine protease inhibitor did not alter LDH release from neutrophils (Fig. 3C,D), whereas inhibition of caspase-1/4 or caspase-3 in unstimulated neutrophils and at MOI 1 significantly reduced the release of LDH (Fig. 3C,D). Taken together, these results suggest that UPEC-evoked IL-1β release from human neutrophils is dependent on NLRP3 and serine proteases and that the release of IL-1β in neutrophils occurs independent of lytic cell death.

### IL-1β release and signaling pathways

**Figure 4:**
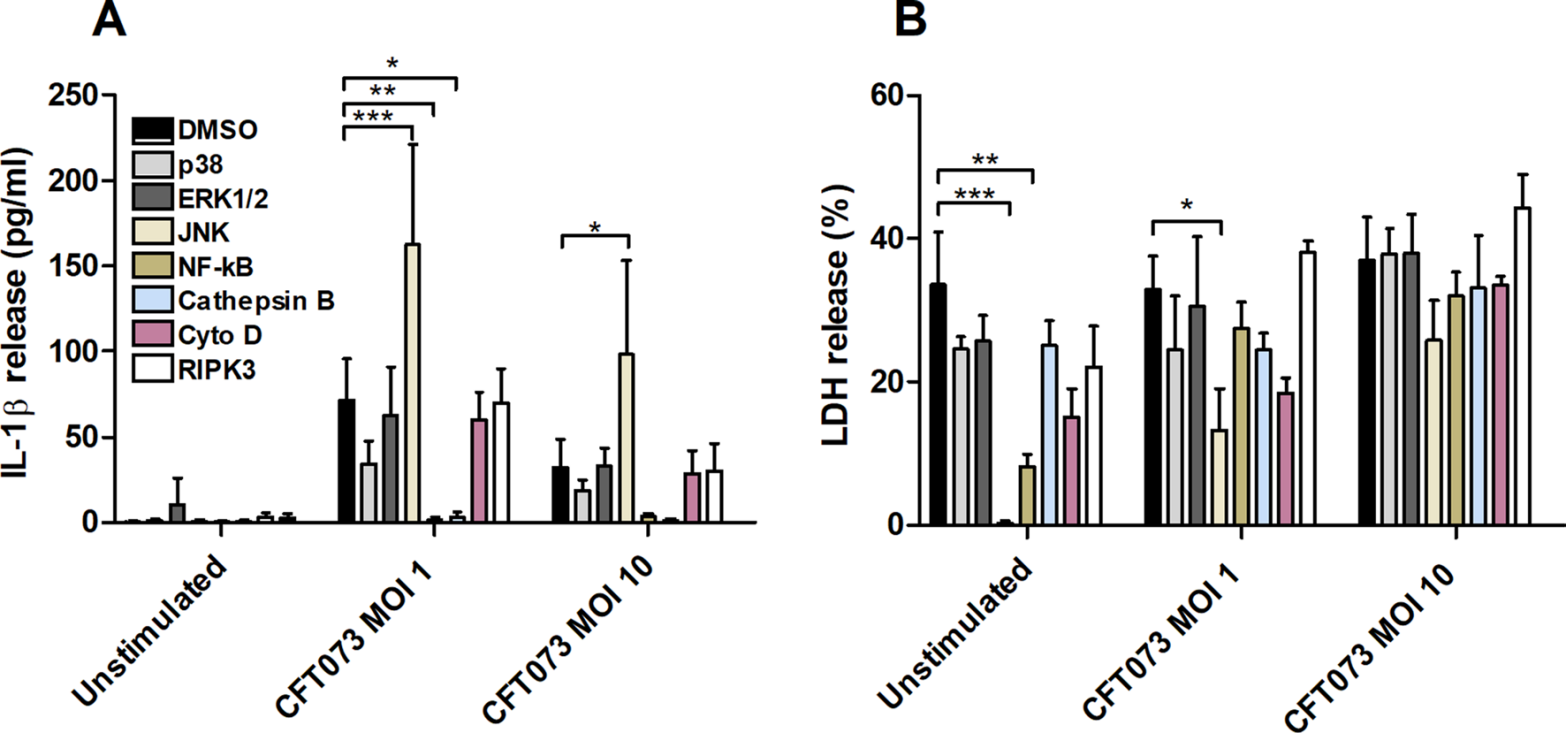
NF-κB and Cathepsin B are associated with UPEC-induced IL-1β release. Neutrophils were pre-incubated with DMSO (vehicle), JNK inhibitor SP600125 (10 µM), p38 MAPK inhibitor SB203580 (10 µM), ERK1/2 inhibitor PD98059 (10 µM), NF-κB inhibitor BAY 11–7082 (5 µM), cathepsin B inhibitor CA074 (100 µM), actin polymerization inhibitor cytochalasin D (Cyto D, 10 µg/ml), RIPK3 inhibitor GSK-872 (10 µM) for 1 h prior to infection with UPEC strain CFT073 for 6 h at MOI 1 or MOI 10 followed by analysis of IL-1β (**A**) and LDH release (**B**). Data are presented as mean ± SEM (n = 3 independent experiments). Asterisks denote statistical significance (* p < 0.05, **p < 0.01, ***p < 0.001).

To investigate the signaling pathways involved in UPEC-induced IL-1β and LDH release from human neutrophils, inhibitors targeting p38, ERK1/2, JNK, NF-κB, cathepsin B, RIPK3 and actin polymerization were used. Inhibition of NF-κB and cathepsin B resulted in a significantly lower release of IL-1β compared to control cells subjected to DMSO, while inhibition of JNK significantly increased UPEC-induced IL-1β release (Fig. 4A). Furthermore, the release of LDH was reduced by the NF-κB and JNK inhibitors in unstimulated cells and by the JNK inhibitor in cells stimulated by UPEC at MOI 1 (Fig. 4B). Together these results suggest that UPEC-induced IL-1β release from human neutrophils is dependent on NF-κB and the cysteine protease cathepsin B.

### UPEC virulence factors and IL-1β release

**Figure 5:**
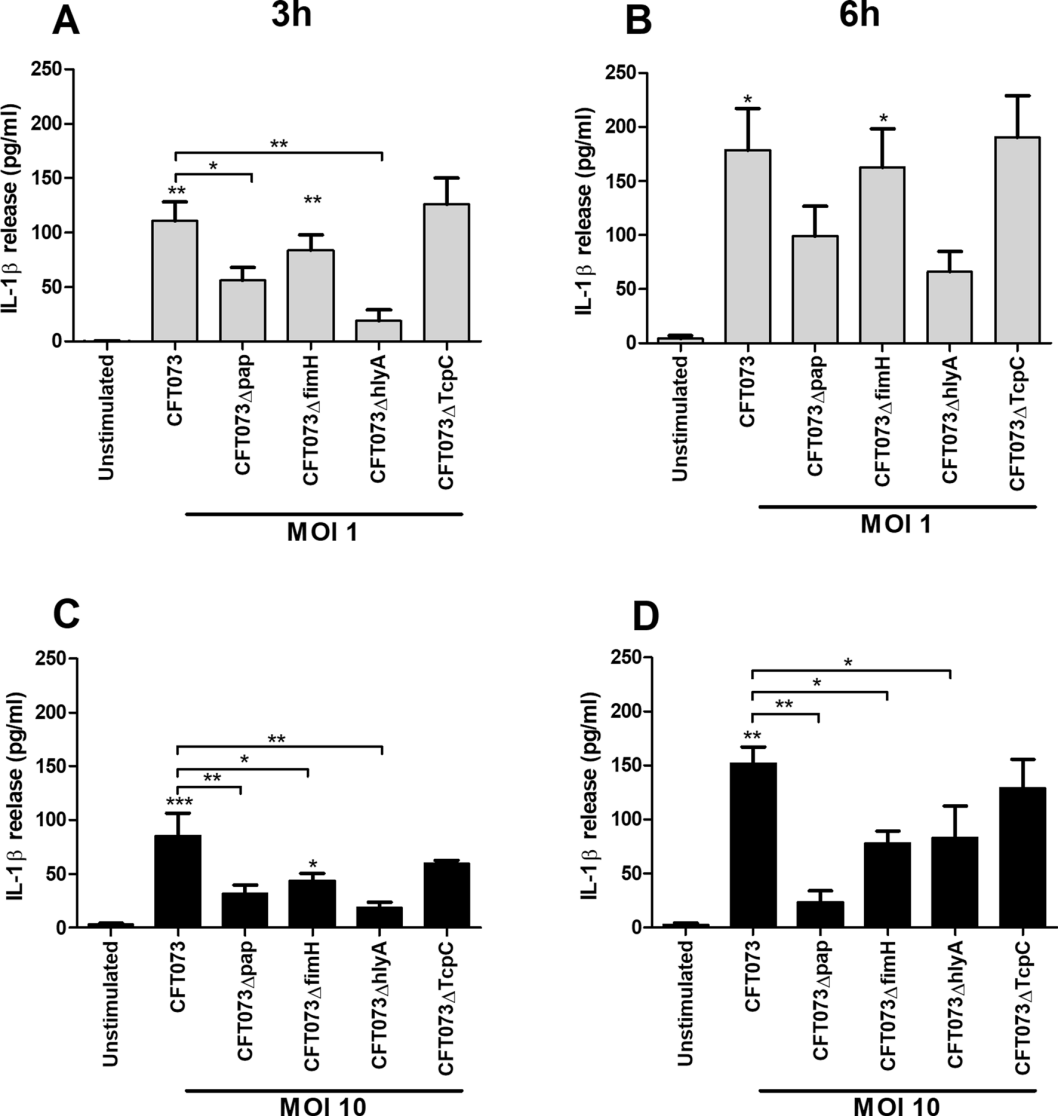
UPEC virulence factors associated with IL-1β release. Neutrophils were infected with UPEC strain CFT073, CFT073Δ*pap*, CFT073Δ*fimH,* CFT073Δ*TcpC* or CFT073Δ*hlyA* at MOI 1 (**A**,**B**) or MOI 10 (**C**,**D**) for 3 h (**A**,**C**) and 6 h (**B**,**D**) followed by analysis of IL-1β release. Data are presented as mean ± SEM (n = 3 independent experiments). Asterisks over bars denote statistical significance compared to respective unstimulated control (*p < 0.05, **p < 0.01, ***p < 0.001).

Experiments were performed to evaluate the involvement of various UPEC virulence factors on IL-1β release using p-fimbriae (*pap*), type-1 fimbriae (*fimH*), α-hemolysin (*hlyA*) or TLR-signaling inhibitory protein (*TcpC*) UPEC deletion mutants. These experiments showed that the Δ*hlyA* and Δ*pap* deletion mutants induced a significantly lower IL-1β release compared to the wild-type UPEC strain at MOI 1 after 3 h (Fig. 5A), but not after 6 h (Fig. 5B). However, at MOI 10 Δ*hlyA*, Δ*pap* and Δ*fimH* deletion mutants induced a significantly lower IL-1β release compared to the wild-type UPEC strain after both 3 and 6 h of stimulation (Fig. 5C,D). The Δ*TcpC* mutant did not differ in its ability to induce IL-1β release compared to wild-type UPEC (Fig. 5A–D). These results suggest that several virulence factors, such as adhesins (p-fimbriae and type-1 fimbriae) and toxins (α-hemolysin), contribute in activation of UPEC-induced release of IL-1β from human neutrophils.

### ROS-production, phagocytosis and the ability of neutrophils to suppress bacterial growth

**Figure 6:**
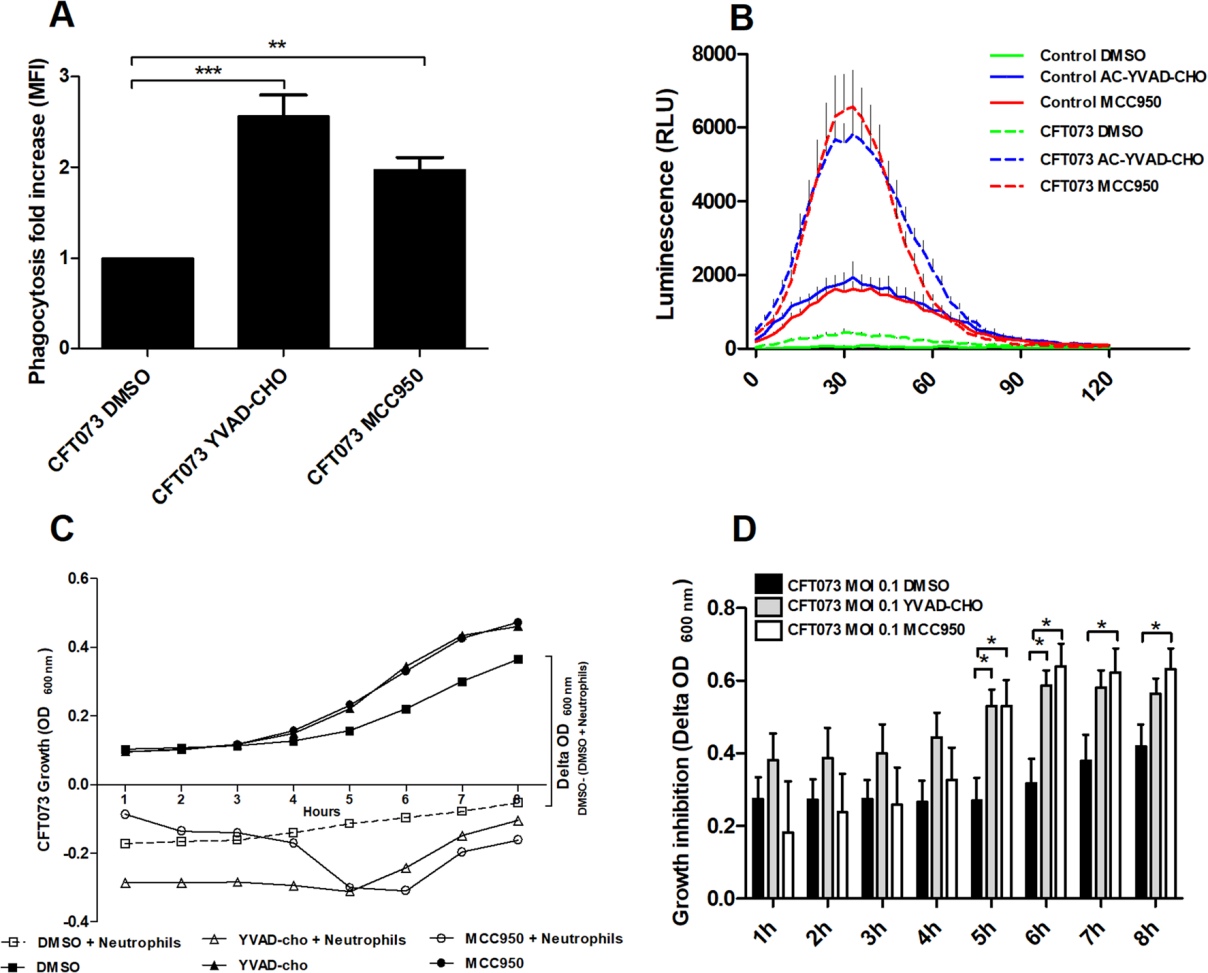
Inhibition of caspase-1/4 and NLRP3 increases antimicrobial properties of neutrophils. Neutrophils were pre-incubated with DMSO (vehicle), caspase-1/4 inhibitor AC-YVAD-CHO (10 µM) or NLRP3 inhibitor MCC950 (2 µM) for 1 h prior to UPEC strain CFT073 infection at MOI 0.1 (**C**,**D**) or MOI 10 (**A**,**B**) for 3 h (**A**), 2 h (B) and 8 h (**C**,**D**) followed by analysis of phagocytosis (**A**), ROS-production (**B**), bacterial growth (**C**) and bacterial growth inhibition (**D**). The relative growth inhibition (delta OD600) was calculated as absorbance of (bacteria + DMSO)—(absorbance of bacteria + neutrophils). Data are presented as mean ± SEM of mean florescence intensity (MFI, **A**), relative luminescence units (RLU, **B**) (n = 3 independent experiments). Asterisks denote statistical significance (*p < 0.05, (**p < 0.01, ***p < 0.001).

We proceeded with studies to assess the contribution of NLRP3 and caspase-1 to the antimicrobial properties of neutrophils. Treatment of neutrophils with the NLRP3 inhibitor MCC950 or the caspase-1/4 inhibitor AC-YVAD-CHO significantly increased the phagocytosis of UPEC compared to neutrophils subjected to the vehicle control DMSO (Fig. 6A). Furthermore, MCC950 and AC-YVAD-CHO significantly increased basal and UPEC-induced ROS production compared to the vehicle control DMSO (Fig. 6B). Next, we examined whether the observed difference in ROS-production and phagocytosis had any effects on growth of UPEC. The bacterial growth response (OD_600_) was inhibited in the presence of neutrophils when compared to bacteria grown in the absence of neutrophils as shown in Fig. 6C. After 5-8 h of co-incubation, the growth inhibition of UPEC was significantly more pronounced in the presence of neutrophils that had been pre-incubated with MCC950 or Ac-YVAD-CHO than in the presence of neutrophils that had been pre-incubated with the vehicle control DMSO (Fig. 6C,D). Taken together, these results suggest that inhibition of caspase-1/4 or NLRP3 increases UPEC-induced neutrophil ROS-production, phagocytosis and the ability to suppress bacterial growth**.**

## Discussion

IL-1β is among the first cytokines to be detectable in urine samples following infection^14^, suggesting early host activation of inflammasomes during UTI. The source of IL-1β during UTI has mainly been associated with activation of the NLRP3 inflammasome in bladder epithelial cells and macrophages^15,17,28^, while less is known about the contribution of neutrophils. In the present study, we demonstrate that UPEC altered the expression of several inflammasome-related genes in neutrophils and that the majority of the genes were down-regulated. It is well-known that UPEC has the ability to suppress host-protective pro-inflammatory responses to favor colonization of the urinary tract^2,4,37^, and IL-18 and NLRP6, previously shown to be important for neutrophil activation^38^ and pyroptosis^39^, were down-regulated by UPEC. Nevertheless, the gene expression of IL-1β and IL-1RA were significantly upregulated by UPEC suggesting that gene activation of the effector molecule IL-1β is still operational despite inhibition of several inflammasome components.

In accordance with the gene expression data, UPEC induced a significant increase of IL-1β release from neutrophils. We also observed that MOI 1 induced a significantly higher IL-1β release compared to MOI 10 after 6 h. This might be linked to the increased cell death observed at MOI 10 compared to MOI 1 after 6 h of infection. Furthermore, UPEC-infection increased protein levels of NLRP3 as well as pro-caspase-1 and activated caspase-1 (p20) in the cell supernatant supporting that UPEC infection activates the NLRP3 inflammasome and caspase-1 in human neutrophils. NLRP3 inflammasome and caspase-1 activation in neutrophils are known to lead to the maturation and release of IL-1β^23,24^. In addition to the classical caspase-1 dependent activation of IL-1β, serine proteases e.g. proteinase 3, neutrophil elastase and cathepsin G have also been found to be involved in the maturation and release of IL-1β from neutrophils by cleaving pro-IL-1β into IL-1β^25,26,30^. The NLRP3 inhibitor MCC950 reduced the UPEC-induced IL-1β release by approximately 50% whereas the serine protease inhibitor DCI essentially eliminated the release of IL-1β. In contrast, the caspase-1/4 and caspase-3 inhibitors increased UPEC-induced IL-1β release. These findings may be explained by the increased viability, measured as LDH release, noted in both unstimulated and stimulated neutrophils in the presence of the caspase-1/4 and caspase-3 inhibitors. Neutrophils are known to undergo a spontaneous cell death as they age, which is important for their homeostasis^40^. Others have shown that inhibiting caspases can delay this spontaneous cell death and retain active neutrophils^40–42^. Inhibition of NLRP3 and serine protease was not accompanied by any effects on lytic cell death, suggesting that the NLRP3- and serine protease-mediated IL-1β release in neutrophils upon UPEC infection takes place independent of lytic cell death. In agreement with our results, others have shown that NLRP3 activation in neutrophils occurs independent of pyroptosis^23^.

**Figure 7:**
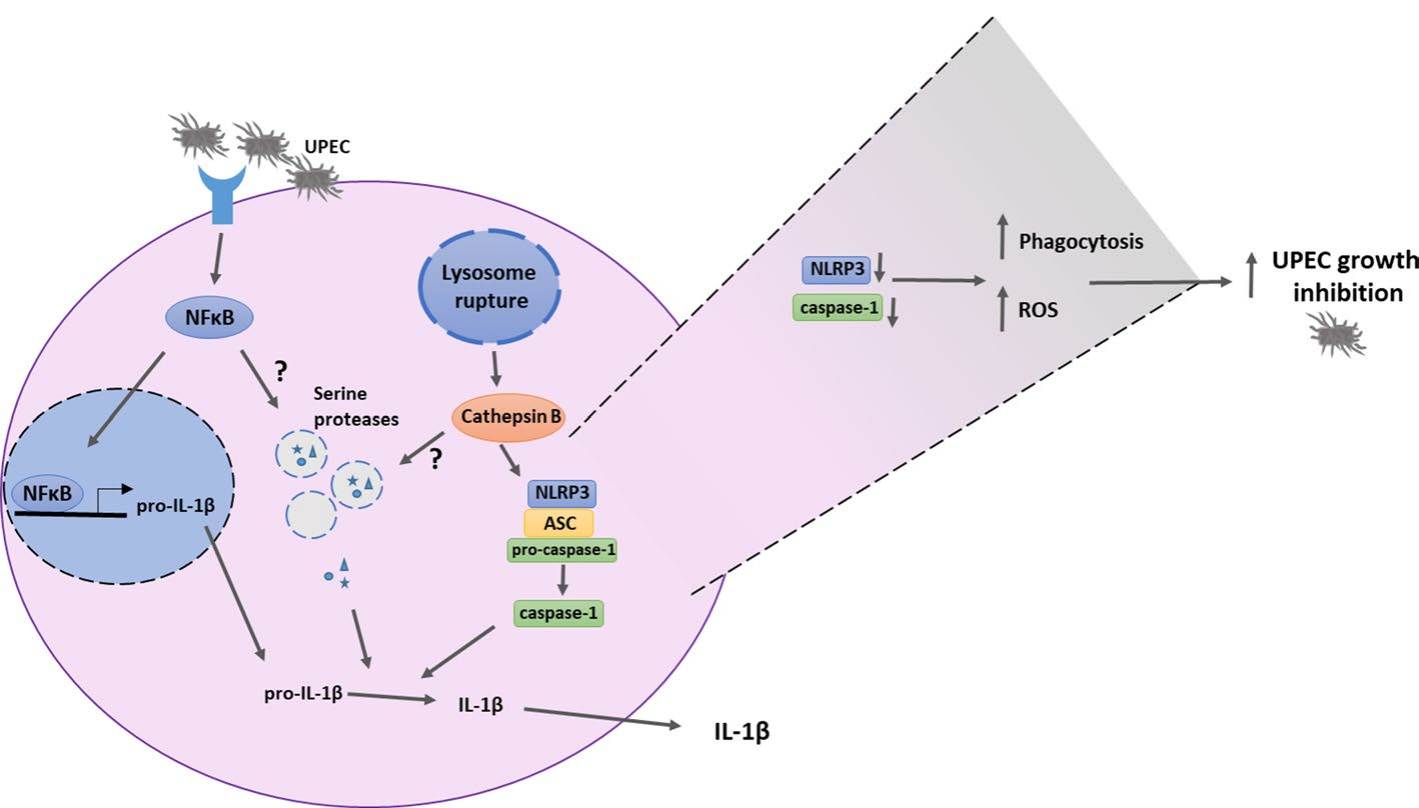
A schematic overview summarizing the main findings. UPEC strain CFT073 induced an increased pro-IL-1β mRNA expression, caspase-1 activation and IL-1β release from human neutrophils. The IL-1β release was mediated by the NLRP3 inflammasome and serine proteases in an NF-κB- and cathepsin B-dependent manner. Additionally, inhibition of NLRP3 and caspase-1 increased neutrophil ROS-production, phagocytosis and the ability of neutrophils to suppress UPEC growth.

A connection between neutrophil serine protease signaling pathways and RIPK3 kinase activity has been implicated, where RIPK3 may induce release of serine proteases from cytoplasmatic granules into the cytosol of neutrophils^43,44^. However, inhibition of RIPK3 did not affect UPEC-induced IL-1β release from neutrophils in our experiments. Further experiments to elucidate signaling pathways for activation of IL-1β release revealed that the release of IL-1β was dependent of NF-κB and cathepsin B, both which are known upstream activators of the NLRP3 inflammasome^21^. This suggests that a priming signal is required for UPEC-mediated IL-1β release from neutrophils. NF-κB-mediated transcriptional priming of pro-IL-1β and NLRP3 is, according to the canonical NLRP3 inflammasome pathway, primarily induced by the engagement of TLR4 by LPS of Gram-negative bacteria^25^. In addition, release of cathepsin B following lysosomal membrane degradation evoked by e.g. ATP, monosodium urate and bacterial components has also been implicated in NLRP3 inflammasome activation^21^. It is not known whether the NF-κB and cathepsin B inhibitors primarily affect NLRP3 activation or down-stream pathways that eventually affect IL-1β release. Based on the Western blot data, the NLRP3 protein was detected in unstimulated neutrophils suggesting a constitutive expression of the NLRP3 inflammasome pathway in neutrophils. Figure 7 illustrates and summarizes the main signaling pathways for UPEC-induced IL-1β release based on results from the present study.

UPEC is known to express numerous virulence factors that can modulate the host immune response to promote colonization of the urinary tract, including the prototypical NLRP3-activating factors LPS and flagellin^2,3^. However, how UPEC virulence factors interact with human neutrophils in terms of IL-1β release is not known. Using type-1 fimbriae (*fimH*), p-fimbriae (*pap*), TLR-signaling inhibitory protein (*TcpC*) and α-hemolysin (*hlyA*) deletion mutants we found that all deletion mutants, except Δ*TcpC*, induced a significantly lower IL-1β release compared to the wild-type UPEC strain. We have previously shown that α-hemolysin, but not type-1 fimbriae or p-fimbriae expression, is crucial for NLRP3 inflammasome activation and IL-1β release from human bladder epithelial cells^15^. This suggests that UPEC-induced IL-1β release in neutrophils is a non-specific and universal response, while the pyroptosis-associated activation of IL-1β release in bladder epithelial cells is more restricted and regulated. Indeed, regulation of inflammation-associated lytic cell death in epithelial cells is highly relevant as exfoliation of bladder epithelial cells may allow access of UPEC to deeper layers of the urothelium and promote its colonization^45^. Typically only one of the type-1 or p-fimbriae types is expressed at a given time, as their expression is phase variable^46,47^. Type-1 fimbriae are expressed by more than 90% of all *E. coli* isolates, both commensal bacteria and UPEC, while between 70 and 90% of all pyelonephritis isolates express the p-fimbriae but only 30% of cystitis isolates^48–51^. α-hemolysin is found among approximately 50% of pyelonephritis and 40% of cystitis strains^49^ and α-hemolysin is known to have several effects on neutrophils. At high concentrations, α-hemolysin induces cell death and at lower concentrations, it is more immunomodulatory^52^. Furthermore, it is interesting that TcpC did not have a suppressive effect on the IL-1β release from human neutrophils. This might be linked to the involvement of serine proteases in the maturation process of IL-1β and not only NLRP3. Collectively, our data suggest that a majority of all UPEC strains will induce the NLRP3-mediated host response in neutrophil and subsequently evoke release of IL-1β. However, the release of IL-1β does not appear to be dependent on the neutrophil phagocytic activity based on the lack of effect of the actin polymerization inhibitor cytochalasin D, known to block phagocytosis^53^. We speculate that neutrophils that arrive at the site of infection have an initiated priming of the NLRP3 inflammasome and serine proteases due to chemotactic gradient migration^54^. The second signal mediated by the bacterial virulence factors e.g. type-1 fimbriae, p-fimbriae and/or α-hemolysin at the site of infection would then promote caspase-1 activation and serine protease release into the cytosol. Once in the cytosol, the serine proteases may induce processing and release of IL-1β from neutrophils.

Neutrophils are critical for UPEC clearance^8^, and experiments performed to address the contribution of inflammasome activation on neutrophil functions of importance for bacterial clearance, revealed that inhibition of NLRP3 and caspase-1/4 increased neutrophil-mediated ROS-production, phagocytosis and growth inhibition of UPEC. Results obtained by Dey et al. have previously shown that bone marrow-derived mice macrophages lacking NLRP3 exhibited increased ROS-production and increased ability to limit *Trypanosoma cruzi* infection compared to wild-type macrophages^55^. NLRP3 knockout macrophages have also been shown to increase phagocytosis of *Neisseria gonorrhoeae*^56^ and deletion of NLRP3 enhanced neutrophil phagocytosis in a polymicrobial sepsis mouse model^57^. The underlying mechanism for the enhanced phagocytosis was found to be associated with increased expression of macrophage receptor with collagenous structure (MARCO) and mannose-binding lectin (MBL) on the surface of NLRP3 knockout neutrophils^57^. These receptors are known to contribute to the uptake of bacteria by neutrophils^57^ and a mechanism involving increased expression of these receptors may also explain our findings. Interestingly, a shift to a NLRP3 deficient phenotype with increased antimicrobial activity may occur during UTI as UPEC have the ability to suppress NLRP3 by the virulence factor TcpC^16^. To the best of our knowledge, this is the first study showing that NLRP3 and caspase-1/4 are involved in neutrophil activation of ROS-production, phagocytosis and IL-1β release following UPEC infection (summarized in Fig. 7). However, a limitation of the present study is that the response of neutrophils to ex vivo UPEC infection may possibly be different from the neutrophil response obtained during in vivo UPEC infection. In vivo studies have shown that IL-1β is part of the innate immune response that protects the urinary tract and counteracts UPEC colonization^16,27^. On the other hand, studies demonstrating a correlation between increased levels of IL-1β and bacterial loads in the mouse bladder^58^, and that IL-1β deficient mice are protected from UTI^20^ rather suggest that IL-1β favors bacterial colonization. Thus, the role of IL-1β in the pathogenesis of UTI is presently unclear. The fact that several cell types in the urinary tract may release IL-1β and a possible processing of IL-1β through inflammasome-independent mechanisms^59^ may contribute to the contradictory results in the literature.

Taken together, UPEC-evoked IL-1β release in neutrophils was found to involve serine proteases and NLRP3. A previously not recognized role of NLRP3 and caspase-1/4 was identified that implicate NLRP3 and caspase-1/4 in the regulation of phagocytosis and ROS-production induced during UPEC infection. Interestingly, UPEC-induced IL-1β release was not associated with lytic cell death pyroptosis in neutrophils, findings in contrast to UPEC-induced IL-1β release in human bladder epithelial cells where α-hemolysin-induced pyroptosis is a prominent feature^15^. Thus, the NLRP3 inflammasome pathway is triggered by UPEC in host cells of the urinary tract although the mechanisms for maturation and release of IL-1β appears to differ depending on cell type.

## Supplementary Information

Supplementary Information.
