# Supplementary material for: Activation of NLRP3 by uropathogenic Escherichia coli is associated with IL-1β release and regulation of antimicrobial properties in human neutrophils
Source: Sci Rep. 2020 Dec 14;10:21837. doi: 10.1038/s41598-020-78651-1 (PMC7736892; doi:10.1038/s41598-020-78651-1)

Supplementary information

**Activation of NLRP3 by uropathogenic *Escherichia coli* is associated with IL-1β release and regulation of antimicrobial properties in human neutrophils**

Isak Demirel^1, 2*^, Alexander Persson^1, 2^, Annelie Brauner^3^, Eva Särndahl^1, 2^, Robert Kruse ^1, 2, 4#^, Katarina Persson^1, 2#^

*^1^ iRiSC - Inflammatory Response and Infection Susceptibility Centre, Faculty of Medicine and Health, Örebro University, Sweden; ^2^ School of Medical Sciences, Örebro University, Örebro, Sweden; ^3^Department of Microbiology, Tumor and Cell Biology, Division of Clinical Microbiology, Karolinska Institutet and Karolinska University Hospital, Stockholm, Sweden. ^4^ Department of Clinical Research Laboratory, Faculty of Medicine and Health, Örebro University, Sweden;*

#Shared last authorship

***Corresponding author**: Isak Demirel PhD, School of Medical Sciences, Campus USÖ, Örebro University, SE-701 82 Örebro, Sweden. Phone: +4619303000. Email: Isak.demirel@oru.se

**Supplementary Table S1*.* Primers used for quantitative real-time PCR.**

| **Gene symbol** | **Oligonucleotide sequences (5´-3´)** |
| --- | --- |
| IL-1β | *F:* CCACAGACCTTCCAGGAGAATG  *R:* GTGCAGTTCAGTGATCGTACAGG |
| *IL-1RA* | *F:* ATGGAGGGAAGATGTGCCTGTC  *R:* GTCCTGCTTTCTGTTCTCGCTC |
| *IL-18* | *F:* GATAGCCAGCCTAGAGGTATGG  *R:* CCTTGATGTTATCAGGAGGATTCA |
| *CASP1* | *F:* GCTGAGGTTGACATCACAGGCA  *R:* TGCTGTCAGAGGTCTTGTGCTC |
| *CASP4* | *F:* GGGATGAAGGAGCTACTTGAGG  *R:* CCAAGAATGTGCTGTCAGAGGAC |
| *CASP5* | *F:* ACAACCGCAACTGCCTCAGTCT  *R:* GAATCTGCCTCCAGGTTCTCAG |
| *PYCARD* | *F:* AGCTCACCGCTAACGTGCTGC  *R:* GCTTGGCTGCCGACTGAGGAG |
| *NLRP1* | *F:* ATTGAGGGCAGGCAGCACAGAT  *R:* CTCCTTCAGGTTTCTGGTGACC |
| *NLRP3* | *F:* GGACTGAAGCACCTGTTGTGCA  *R:* TCCTGAGTCTCCCAAGGCATTC |
| *NLRC4* | *F:* AGGTCCCACAACTCGTCAAGCT  *R:* TGCTCACACGATTTCCCGCCAA |
| *NLRP6* | *F:* ACTGTGCCATCTGAGCAGCCTC  *R:* TCACTGAGCCTGTTGTGGAGGA |
| *AIM2* | *F:* GCTGCACCAAAAGTCTCTCCTC  *R:* CTGCTTGCCTTCTTGGGTCTCA |
| *GAPDH* | *F:* GTCTCCTCTGACTTCAACAGCG  *R:* ACCACCCTGTTGCTGTAGCCAA |

**Supplementary Figure S1**

**Supplementary Figure S1:** Original whole western blots shown. The pro-capsase-1, caspase-1 p20, GAPDH membranes were cut before antibody probing. This was done in order to probe for several proteins simultaneously. Pro-caspase-1 and Caspase-1 p20 from supernatant are the same blot with different exposure times.

**CFT073 MOI10**

**Unstimulated**

**CFT073 MOI1**


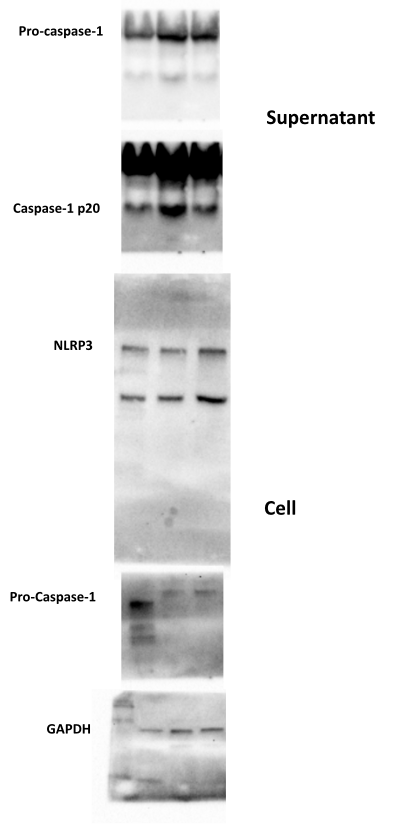

Supplement: Supplementary file 1 — Supplementary Information. [file 41598_2020_78651_MOESM1_ESM.docx]
